# Early Toxicities After High Dose Rate Proton Therapy in Cancer Treatments

**DOI:** 10.3389/fonc.2020.613089

**Published:** 2021-01-14

**Authors:** Jérôme Doyen, Marie-Pierre Sunyach, Fabien Almairac, Véronique Bourg, Arash O. Naghavi, Gwenaëlle Duhil de Bénazé, Audrey Claren, Laetitia Padovani, Karen Benezery, Georges Noël, Jean-Michel Hannoun-Lévi, Ferran Guedea, Jordi Giralt, Marie Vidal, Guillaume Baudin, Lucas Opitz, Line Claude, Pierre-Yves Bondiau

**Affiliations:** ^1^ Université Côte d’Azur, Department of Radiation Oncology, Centre Antoine-Lacassagne, Fédération Claude Lalanne, Nice, France; ^2^ Department of Radiotherapy, Léon Bérard Cancer Center, Lyon, France; ^3^ Department of Neurosurgery, Centre Hospitalier Universitaire, University Côte d’Azur, Nice, France; ^4^ Department of Neurology, Centre Hospitalier Universitaire, University Côte d’Azur, Nice, France; ^5^ Department of Radiation Oncology, H. Lee Moffitt Cancer Center and Research Institute, Tampa, FL, United States; ^6^ Department of Pediatric Oncology, Centre Hospitalier Universitaire, University Côte d’Azur, Nice, France; ^7^ Department of Radiation Oncology, Centre Antoine-Lacassagne, Nice, France; ^8^ Oncology Radiotherapy Department, CRCM Inserm, UMR1068, CNRS UMR7258, AMU UM105, Genome Instability and Carcinogenesis, Assistance Publique des Hôpitaux de Marseille, Aix-Marseille University, Marseille, France; ^9^ Department of Radiation Oncology, Institut de cancérologie Strasbourg Europe (Icans), Strasbourg, France; ^10^ Radiation Oncology Department, Institut Català d’Oncologia (ICO) and University of Barcelona (UB), L’Hospitalet de Llobregat, Barcelona, Spain; ^11^ Hospital Vall d’Hebron, Vall d’Hebron Institute of Oncology, Barcelona, Spain; ^12^ Department of Radiology, Centre Antoine-Lacassagne, Nice, France; ^13^ Department of Anesthesiology, Centre Antoine-Lacassagne, Nice, France

**Keywords:** high dose rate, proton therapy, cancer, early, subacute, toxicity

## Abstract

**Background:**

The conventional dose rate of radiation therapy is 0.01–0.05 Gy per second. According to preclinical studies, an increased dose rate may offer similar anti-tumoral effect while dramatically improving normal tissue protection. This study aims at evaluating the early toxicities for patients irradiated with high dose rate pulsed proton therapy (PT).

**Materials and Methods:**

A single institution retrospective chart review was performed for patients treated with high dose rate (10 Gy per second) pulsed proton therapy, from September 2016 to April 2020. This included both benign and malignant tumors with ≥3 months follow-up, evaluated for acute (≤2 months) and subacute (>2 months) toxicity after the completion of PT.

**Results:**

There were 127 patients identified, with a median follow up of 14.8 months (3–42.9 months). The median age was 55 years (1.6–89). The cohort most commonly consisted of benign disease (55.1%), cranial targets (95.1%), and were treated with surgery prior to PT (56.7%). There was a median total PT dose of 56 Gy (30–74 Gy), dose per fraction of 2 Gy (1–3 Gy), and CTV size of 47.6 ml (5.6–2,106.1 ml). Maximum acute grade ≥2 toxicity were observed in 49 (38.6%) patients, of which 8 (6.3%) experienced grade 3 toxicity. No acute grade 4 or 5 toxicity was observed. Maximum subacute grade 2, 3, and 4 toxicity were discovered in 25 (19.7%), 12 (9.4%), and 1 (0.8%) patient(s), respectively.

**Conclusion:**

In this cohort, utilizing high dose rate proton therapy (10 Gy per second) did not result in a major decrease in acute and subacute toxicity. Longer follow-up and comparative studies with conventional dose rate are required to evaluate whether this approach offers a toxicity benefit.

## Introduction

Proton Therapy (PT) is a particle therapy that utilizes a Bragg Peak to reduce the radiation dose received by healthy tissue, as demonstrated by previous *in silico* studies (1). PT is preferred in patients with a long-life expectancy, to mitigate the risk of late sequela (e.g., secondary malignancy, cardiovascular complications, etc.) or in patients where the ideal dose is difficult to achieve without a significant toxicity risk. Several studies have demonstrated a clinical advantage of PT over conventional photon radiotherapy (2–6), with many prospective clinical trials ongoing.

To further improve the therapeutic ratio, several preclinical studies identified a considerable biological advantage to delivering radiation dose rate higher than the conventional 0.01 to 0.05 Gy/second. “FLASH” radiotherapy, or dose rates exceeding 10 Gy per 100 ms, significantly reduced the radiation damage to healthy cells/tissue without a decline in anti-tumoral effect (7–9). This was initially demonstrated with electrons (7–9), but subsequently with photons (10) and protons (11–13). There is a scarcity of clinical data utilizing FLASH radiotherapy, with only 1 case report to date, which showed that electron FLASH reirradiation may mitigate toxicity and allow radiation delivery even if the theoretical cumulative doses to healthy tissue would be exceeded (14).

A recent clinical device (Proteus One^©^, Ion Beam Application) was designed to deliver pencil-beam scanning with pulsed proton at high dose rate, approximately 200–1,000 times faster than the classical dose rate (125 million protons per pulse leading to approximately 10 Gy/s per spot, depending on the range and energy needed) (15). Toxicity with this dose rate level per spot has not yet been reported.

The purpose of the current study is to analyze the early toxicities for tumors treated pencil-beam scanning with pulsed high dose rate protons.

## Materials and Methods

### Patient Selection

With institutional review board approval, we retrospectively analyzed patients receiving PT at our institution between September 2016 and April 2020. This study included patients that were treated for a benign or malignant tumor, had at least 3 months of clinical follow-up, and received radiation with only PT (without a photon component). Reirradiation was included, defined as an overlap in the previous field with the current GTV. Patients addressed from other centres were not included because of the lack of updated follow-up.

### Follow-Up

Patients were followed weekly during PT or more frequently if necessary. A 1-month clinical follow-up was performed after the completion of PT. Benign tumors underwent magnetic resonance imaging (MRI) and clinical follow-up 4 months after completing PT and every 6 months thereafter. Malignant tumors underwent imaging according to the standard of care for that malignancy, which included an MRI of the irradiated region and clinical follow-up every 4 months for 2 years, then every 6 months for 3 years, and a computed tomography (CT) scan of the thorax and abdomen if required (e.g., head and neck cancer, sarcomas, etc.).

Tumor response was evaluated according to RECIST (response criteria in solid tumors) 1.1 criteria (16) and toxicities according to the fifth version of the Common Terminology Criteria for Adverse Events (CTCAE). Toxicity was considered “acute” if occurred during PT or within 2 months of completing PT, and “subacute” if occurred >2 months after completing PT. Late toxicity was not reported due to short follow-up.

### Proton Therapy

PT was delivered with Pencil Beam Scanning (PBS), utilizing the Proteus One^©^ device (Ion Beam Application^©^), which is a synchrocyclotron with active pencil beam scanning and pulsed beam PT. Approximately 125 million protons are delivered per pulse/spot with an energy of 100 to 226 MeV as a function of the target depth. The dose rate per spot is approximately 10 Gy per second, depending on the energy. The characterization of this beam was previously reported by Rossomme et al (15). PT immobilization was performed with a thermoplastic mask for head and neck targets or vacuum body cast for extracranial targets.

The dosimetry was performed using the Raystation treatment planning system ^©^ version 6.0 before June 2019 and version 8.0 after June 2019 (Raysearch Laboratories, Stockholm, Sweden). Dose constraints from Feuvret et al (17). and by Marks et al (18). were used for head/neck and extracranial targets, respectively. A relative biologic effectiveness factor for protons of 1.1 was incorporated. PBS plans were calculated using robust optimization (3% range uncertainties-3 mm positioning uncertainties, for cranial target and 3%–5 mm for extracranial target). Robustness was applied for CTV, brain stem, spinal cord, optic nerves, optic chiasm, femoral head, and digestive tract.

### Statistical Analysis

Tumor response was defined by progression, stabilization, partial response, or complete response. Time-to-event outcomes were estimated from the date of last PT fraction to an event or censored at last follow up. This included local control (LC), and progression-free survival (PFS). LC and PFS were evaluated *via* Kaplan-Meier method with a 95% confidence interval (IC 95%). Median follow-up was evaluated by the Schemper method. All statistical analyses were performed using Statistical Package for the Social Sciences (SPSS) version 16.0 on Windows^®^.

## Results

### Characteristics of Patients and Treatments

Characteristics of patients and treatments are described in **Table 1**. A total of 127 patients were included. This cohort most commonly consisted of benign disease (55.1%), head and neck location (95.1%), and ECOG ≤ 1 (95.8%). Majority of patients had treatments prior to PT, which included surgery (56.7%), chemotherapy (26%), and previous radiotherapy to the same location (14.2%). Two patients were irradiated with a bifractionated schedule because of previous radiation.

**Table 1:** Patient and treatment characteristics.

Variable	
**Median age (years)**	55 (1.6–89)
**Gender**	
Male	59 (46.5%)
Female	68 (53.5%)
**Histology**	
**Benign brain tumors**	
Meningioma	34 (26.7%)
Schwannoma	4 (3.2%)
Paraganglioma	4 (3.2%)
Craniopharyngioma	4 (3.2%)
Pituitary adenoma	7 (5.5%)
Primary orbitary tumors (lymphoma)	2 (1.6%)
Benign vascular tumor	3 (2.4%)
**Malignant brain tumors**	
Ependymoma	4 (3.2%)
Low grade glioma	13 (10.2%)
**Bone tumors**	
Chordoma	10 (7.9%)
Chondrosarcoma	3 (2.4%)
Giant cell tumor	1 (0.7%)
Ewing sarcoma	3 (2.4%)
**Malignant head and neck tumors**	
Malignant paranasal and nasal sinus tumors	14 (11%)
Salivary gland carcinoma	6 (4.7%)
**Sarcoma**	
Rhabdomyosarcoma	6 (4.7%)
Sarcoma	5 (3.9%)
**Other**	
Merckel carcinoma	1 (0.7%)
Isolated/local relapse of other cancer*	3 (2.4%)
**Tumor location**	
Head and neck	111 (95.1%)
Pelvis	14 (4.3%)
Paraspinal	2 (0.6%)
**Previous surgery**	
Yes	72 (56.7%)
No	55 (43.3%)
**Previous chemotherapy**	
Yes	33 (26%)
No	94 (74%)
**Reirradiation setting**	
Yes	18 (14.2%)
No	109 (85.8%)
**Concomitant chemotherapy**	
Yes	13 (10.2%)
No	114 (89.8%)
Performance Status ECOG**	
At the beginning of Proton Therapy	
0	79 (62.7%)
1	42 (33.1%)
2	5 (3.9%)
Missing	1 (0.8%)
**Radiotherapy**	
Median residual tumor volume	12.5 ml (0–672)
Median low-risk CTV	47.6 ml (5.6–2106.1)
Median high-risk CTV (if boost, n=46)	35.3 ml (4.1–560.9)
Median dose per fraction	2 Gy (1–3)
Median number of fractions	30 (12–60)
Median total dose	56 Gy (30–74)
Median PT duration	48 days (18–82)
Bifractionated	2 (1.6%)

*Rectal cancer, vaginal squamous cell carcinoma, clear cell carcinoma of the kidney.

**Eastern Cooperative Oncology Group.

The most common diseases in this study include: meningioma (10.4%), followed by malignant paranasal and nasal sinus tumors (4.3%), and low-grade gliomas (4%).

Chemotherapy was also delivered concomitantly with PT (10.2%) and adjuvant after PT (11%). Concomitant chemotherapy was used for Ewing sarcoma (n=3, vincristine, doxorubicin, ifosfamide), rhabdomyosarcoma (n=6, ifosfamide, vincristine), malignant sinus tumor (n=3, platinum-based chemotherapy), and metastatic lymph node from a vulvar squamous cell carcinoma (n=1, platinum-based chemotherapy). After PT, 1 patient underwent surgical resection.

Among patients with meningioma, malignant sinus tumor and low-grade gliomas, the median total PT dose delivered was 56 Gy (54–60), 68.2 Gy (45–70.4), and 55.8 Gy (54–60), with a median dose per fraction of 2 Gy (1.8–2), 2 Gy (1.8–3), and 2 Gy (1.8–2), and a median CTV size of 41.4 ml (6.7–250.9), 88.2 ml (22.1–572.8), and 65.5 ml (6.2–422.1), respectively.

Among patients with pelvic (n=14) or paraspinal tumors (n=2) the median total PT dose was 65.1 Gy (50.4–73.5), with a median dose per fraction of 2 Gy (1.8–2.4), and a median low risk and high risk CTV size of 437.4 ml (83.2–2,106) and 112.5 ml (14.5–560.9), respectively.

Median follow-up was 14.8 months (3;-42.9). Locally, 5 (3.9%) patients experienced a complete response, 31 (24.4%) a partial response, 71 (55.9%) stabile disease, 5 (3.9%) progressive disease (PD), and 17 (11.9%) did not relapse after combine combination of surgery and PT (local control). The 1-year local control and progression-free survival were 89.2% and 85%, respectively.

### Acute Toxicities

Acute toxicities are defined as side-effects that occur during PT or within 2 months of completing PT (**Table 2**). Maximum acute toxicity grade was 0, 1, 2, and 3 for 20 (15.7%), 58 (45.7%), 41 (32.3%), and 8 (6.3%) patients, respectively. There were no grade 4 or 5 acute toxicities.

**Table 2:** Acute toxicities.

	Grade 1	Grade 2	Grade 3	Grade 4	Total
Alopecia	40	0	0	0	40
Dermatitis radiation	24	14	1	0	39
Asthenia	20	6	0	0	26
Headache	20	3	1	0	24
Dry eye	18	6	0	0	24
Nausea	14	3	1	0	18
Sinusitis	8	7	0	0	15
Oral mucositis	9	1	2	0	12
Local pain	6	5	1	0	10
Dysgeusia	6	3	0	0	9
Dry mouth	5	2	0	0	7
Dizziness	5	0	0	0	5
Orbit edema	4	1	0	0	5
Anosmia	5	0	0	0	5
Seizure	1	2	1	0	4
Trismus	4	0	0	0	4
Local bleeding	4	0	0	0	4
Dyspnea	1	2	0	0	3
Diarrhea	3	0	0	0	3
Hearing impaired	2	0	0	0	2
Cranial nerve disorder	0	0	2	0	2
External otitis	2	0	0	0	2
Dysesthesia	1	1	0	0	2
Keratitis	1	0	0	0	1
Esophagitis	1	0	0	0	1
Palpitations	1	0	0	0	1
Cystitis	1	0	0	0	1
Vomiting	0	2	0	0	1

**Figure 1:**
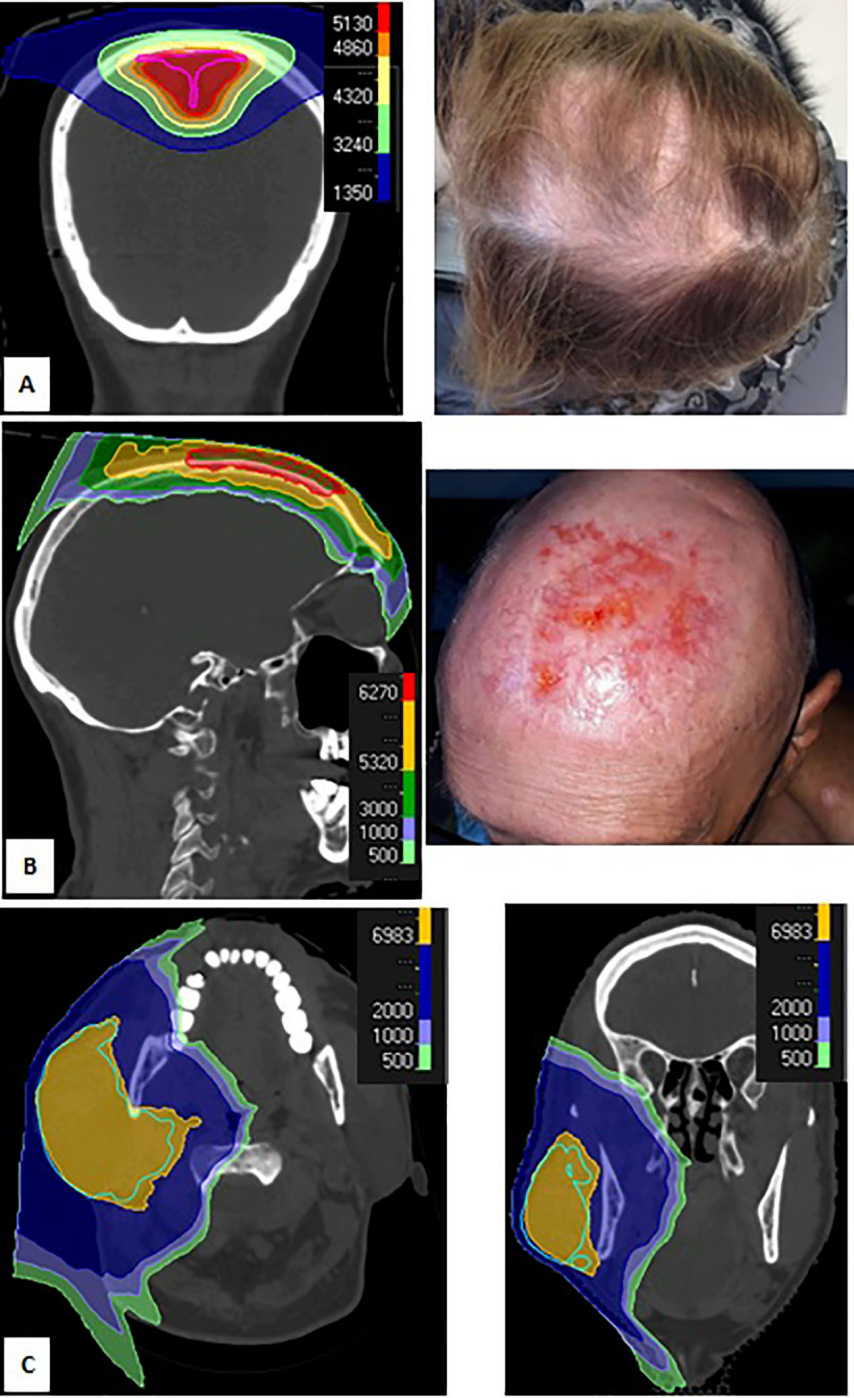
Dose distribution (**A,** left) and grade 1 alopecia (**A,** right) during proton therapy (PT) of patient with meningioma; dose distribution (**B,** Left) and grade 3 dermatitis (**B**, right) 1 month after PT for Merckel carcinoma of the vertex; dose distribution for cystic adenoid carcinoma of the right parotid (**C,** right, axial view; **C,** left, coronal view).

The most frequent acute toxicity was alopecia (n=40), primarily in targets close to skin or eyelids (88 patients). **Figure 1A** describes an example of alopecia occurring during PT in a patient treated for meningioma (Patient 1). The second most frequent acute toxicity was radiation dermatitis (n=39), which occurred when irradiating close to the skin surface. An example is a vertex angiosarcoma patient treated to 66 Gy (2 Gy per fraction, no concomitant chemotherapy) (**Figure 1B**), who presented with a grade 3 dermatitis at 46 Gy, which slowly healed 1 month after completing PT (Patient 2). Of note 10 out of 13 patients irradiated with concomitant chemotherapy presented with dermatitis.

Only one patient with a head and neck tumor received radiation to their primary and bilateral lymph nodes, whereas all other patients received radiation to only their primary or ipsilateral neck (e.g., salivary gland tumors). **Figure 1C** describes a 79 year-old patient with non-operable cystic adenoid carcinoma irradiated to 73.5 Gy (2.1 Gy per session) (Patient 3). This treatment was well tolerated with only grade 1 oral mucositis and no dysgeusia.

In pelvic and paraspinal tumors (n=16), PT was well tolerated with no grade ≥ 3 gastrointestinal toxicities. Of note, there were only 4 grade 1 gastrointestinal toxicities in this subgroup (3 diarrhea and 1 nausea).

### Subacute Toxicities

Subacute toxicity, which occurred >2 months after completing PT, are detailed in **Table 3**. Maximum subacute toxicity grade was 0, 1, 2, 3, and 4 for 50 (39.4%), 39 (30.7%), 25 (19.7%), 12 (9.4%), and 1 (0.8%) patient(s), respectively. There were no grade 5 toxicities.

**Table 3:** Subacute toxicities.

	Grade 1	Grade 2	Grade 3	Grade 4	Total
Dry eye	16	4	0	0	20
Seizure	4	3	6	0	13
Headache	11	2	0	0	13
Alopecia	12	0	0	0	12
Asthenia	6	5	0	0	11
Sinusitis	4	5	0	0	9
Dry mouth	5	0	0	0	5
Skin pigmentation	5	0	0	0	5
Dizziness	4	0	0	0	4
Orbit edema	3	1	0	0	4
Hearing impaired	1	3	0	0	4
Local pain	2	1	0	0	3
Dermatitis	2	1	0	0	3
Cranial nerve disorder	1	2	0	0	3
Neuropathy	1	2	0	0	3
Dysgeusia	2	0	0	0	2
Trismus	2	0	0	0	2
Brain radionecrosis	1	1	0	0	2
External otitis	2	0	0	0	2
Pulmonary embolism	0	0	2	0	2
Local bleeding	1	0	1	0	2
Brain stroke	2	0	0	0	2
Optic nerve disorder	0	0	0	1	1
Cataract	0	1	0	0	1
Nausea	1	0	0	0	1
Fracture	0	0	1	0	1
Cystitis	1	0	0	0	1
Mastoiditis	0	0	1	0	1
Dysphagia	1	0	0	0	1
Dysphonia	1	0	0	0	1
Dyspnea	1	0	0	0	1
Colonic obstruction	0	0	1	0	1
Cough	1	0	0	0	1
Depression	1	0	0	0	1
Memory loss	1	0	0	0	1

The most common subacute toxicity was dry eye (n=20, 18% among cranial targets, no grade ≥3 toxicity), followed by seizures (n=14, 12.6% among cranial targets). All patients with grade 3 seizures (n=6) were due to a new onset seizure with no prior seizure history. Seizures were medically manageable for all patients. Histology of patients who presented with a subacute onset seizure was as follows: meningioma (n=7), low grade glioma (n=4), ependymoma (n=1) and pituitary adenoma (n=1). Of these, 8 presented with baseline seizures, 7 of which were on antiepileptic drugs. Median delay to new onset or worsening of seizure was 4.6 months (0.8–34.2) from the end of PT. Of the pelvic and primary tumors, there was 1 grade 3 toxicity (colonic obstruction), in a previously irradiated pelvic sarcoma, requiring hospitalization and resolved with medical management. There was 1 grade 4 toxicity (left optic nerve disorder), which occurred in a 72-year old women treated for skull-based meningioma surrounding optic nerves bilaterally. Left blindness occurred 1 year after PT with no evidence of relapse. D1, D2 and Dmean to the left optic nerve were 53.7 Gy, 53.7 Gy, and 53.3 Gy, respectively.

## Discussion

To our knowledge the current study is the first to report early outcomes after high dose rate pulsed proton therapy. The cohort primarily consists of cranial targets and benign disease. The acute toxicities were as expected according to dosimetry, especially for acute onset alopecia and dermatitis.

Unfortunately, the use of high dose rate pulsed PT did not provide a significant “FLASH-like” radioprotective effect.

According to the FLASH electron therapy data, we should have observed a much lower rate and intensity of acute side-effects. In the preclinical setting, Favaudon et al. delivered 15 Gy in a single FLASH dose (60 Gy/s) to the whole lungs without inflammatory infiltration or extracellular matrix deposition after 62 days, whereas mice irradiated with classical dose rates (0.03/s) presented with dense inflammatory infiltrate and extracellular matrix deposition (7).

In our cohort, no patient received lung irradiation and cannot comment on whether our “FLASH effect” might be dependent on the nature of the irradiated tissue. However, Montay-Gruel et al. reported that FLASH photon therapy could also have a protective effect during brain irradiation (8, 10). Unfortunately, we observed seizure rates of 3.6% (n=4 of 111 patients with cranial irradiation) during PT and 12.6% (n=14 of 111 patients with cranial irradiation) 2 months after PT. Most patients had a previous history of epilepsy (n=8) and we observed six new epilepsy diagnosis after PT. Weber et al. reported a similar rate of post-radiation seizure after radiotherapy (21.5%) for atypical and malignant meningioma (EORTC 22042–26042 phase II study) (19). Lynam et al. observed a cumulative “delayed” seizure (after treatment) rate of 33.8% (n=22 of 65 patients), especially in patients with low grade glioma or meningioma, as seen in our study (20).

We observed a very favourable toxicity profile for Patient 3 treated for non-operable cystic adenoid carcinoma of the parotid (**Figure 1C**). Romesser et al. previously reported that ipsilateral head and neck PT is associated with a dramatic decrease in the rate of acute toxicities when compared with photon therapy, such as grade ≥ 2 dysgeusia (5.6% vs 65.2%, p<0.001) and grade ≥ 2 mucositis (16.7% vs 52.2%, p=0.01) (21). The favorable toxicity profile observed for Patient 3 is likely related to the Bragg Peak advantage. In contrast, Patient 2 was treated for skin cancer of the scalp vertex and presented with prolonged grade 3 toxicity. This is consistent with the unfavorable skin tolerance seen in previously described breast cancer cohorts treated with conventional dose rate PT, with a rapid dose deposit on the skin surface, which may be mitigated with proper dose constraints (22).

Despite the high dose delivered (median of 65.1 Gy [50.4–73.5]) and large irradiated volume (median of 437.4 ml [83.2–2106]), there were limited gastrointestinal sequela for the 16 pelvic and paraspinal tumors, with only 1 grade 3 subacute toxicity (digestive occlusion) reported 4 months after re-irradiation for an 89 year old pelvic sarcoma (65.1 Gy in 31 fractions). This patient required hospitalization but the occlusion quickly resolved with medical management alone. This is consistent with the study by Schneider et al, who described the conventional dose rate PT of 31 paraspinal/retroperitoneal patients, with a mean total dose of 72.3 Gy in 1.8–2 Gy fractions and planning target volume of 560.2 ml, observed no acute or late grade ≥ 2 toxicities (23). Similar to the favorable outcome in Patient 3, toxicity profile of the pelvic/paraspinal cohort is likely due to the Bragg Peak advantage rather than a FLASH-like effect.

Limitations of this study include: its retrospective nature, the relative small number of patients, and short follow-up. With the toxicities (e.g., dermatitis, alopecia, and seizures) observed in this study, we can conclude that the use of high dose rate proton therapy (around 10 Gy/s per spot) does not offer the expected FLASH-like radioprotective effect. It may cause lower grade toxicities but proper comparative studies with conventional dose rate PT are needed. The dose rate might be dependent on the type of irradiation and may be more difficult to obtain with protons than it is for photons or electrons. Larger dose per fraction may also be needed. The FLASH effect of protons was analyzed in the preclinical setting by Buonanno et al, who evaluated various dose rate (0.05, 10, 20, 100, and 1000 Gy/s) effect on normal lung fibroblasts. They found that the proton dose rate had little impact on acute effects, but favorably influenced the 1-month expression of TGF-β (inverse expression with dose rate) and 1-month cell senescence (lower senescence with higher dose rate) (12). Therefore, the FLASH effect of protons might be less pronounced than with other particles and may require an even higher dose rate to be clinically significant. Of note, most of this cohort consisted of head and neck tumors, where toxicity benefit of high dose rate proton therapy may not be as significant as other disease sites, such as abdominal or thoracic treatment sites, which will require further analysis in future studies.

In conclusion, the present study describes the early outcomes with use of high dose rate proton therapy (around 10 Gy/s). Contrary to what was expected in preclinical studies, there was no FLASH-like effect (no lack of toxicities). To identify a clinical difference, when compared to the classical dose rate (<0.05 Gy/s), may require larger cohorts, a match-paired retrospective study or randomized prospective study, longer follow up, or possibly a higher dose rate or dose per session.

## Data Availability Statement

The raw data supporting the conclusions of this article will be made available by the authors, without undue reservation.

## Ethics Statement

This study requires a declaration to our French administration (CNIL) and the information of patients by using an information letter. It was performed and the declaration number is as following: 2204177 v 0.

## Author Contributions

JD and P-YB conceptualized and designed the study. JD and P-YB acquired, analyzed and interpreted the data. JD, M-PS, FA, VB, AN, GB, LP, J-MH-L, FG, JG, MV, AC, KB, LC, and P-YB drafted/revised the work for intellectual content and context. JD gave the final approval and overall responsibility for the published work. All authors contributed to the article and approved the submitted version.

## Conflict of Interest

The authors declare that the research was conducted in the absence of any commercial or financial relationships that could be construed as a potential conflict of interest.

## Abbreviations

CT, computed tomography; CTCAE, Common Terminology Criteria for Adverse Events; CTV, clinical target volume; GTV, gross tumor volume; IMPT, intensity modulated proton therapy; LC, local control; LGG, low grade glioma; MRI, magnetic resonance imaging; PBS, Pencil Beam Scanning; PFS, progression-free survival; PT, Proton Therapy; PTV, planning target volume; RECIST, response criteria in solid tumors.
